# Auditory capacity of the better-hearing ear in asymmetric hearing loss

**DOI:** 10.1007/s00405-023-08342-w

**Published:** 2023-11-25

**Authors:** Iva Speck, Elisabeth Gundlach, Sandra Schmidt, Nadine Spyckermann, Anke Lesinski-Schiedat, Ann-Kathrin Rauch, Antje Aschendorff, Kruthika Thangavelu, Katrin Reimann, Susan Arndt

**Affiliations:** 1 Department of Otorhinolaryngology - Head and Neck Surgery, Medical Center, Faculty of Medicine, University of Freiburg, Killianstr. 5, 79110 Freiburg, Germany; 2 Department of Oto-Rhino-Laryngology, Central Army Hospital Koblenz, Ruebenacher Str. 170, 56072 Koblenz, Germany; 3 Department of Otorhinolaryngology, Medical University Hannover, Carl-Neuberg-Str. 1, 30625 Hannover, Germany; 4 Department of Otorhinolaryngology, Head and Neck Surgery, University Hospital Marburg, Philipps-University Marburg, Marburg, Germany

**Keywords:** SSD, Single-sided deafness, Unilateral hearing loss, AHL, Asymmetric hearing loss

## Abstract

**Purpose:**

Our aim was to investigate the course of the hearing capacity of the better-hearing ear in single-sided deafness (SSD) and asymmetric hearing loss (AHL) over time, in a multicenter study.

**Methods:**

We included 2086 pure-tone audiograms from 323 patients with SSD and AHL from four hospitals and 156 private practice otorhinolaryngologists. We collected: age, gender, etiology, duration of deafness, treatment with CI, number and monosyllabic speech recognition, numerical rating scale (NRS) of tinnitus intensity, and the tinnitus questionnaire according to Goebel and Hiller. We compared the pure tone audiogram of the better-hearing ear in patients with SSD with age- and gender-controlled hearing thresholds from ISO 7029:2017.

**Results:**

First, individuals with SSD showed a significantly higher hearing threshold from 0.125 to 8 kHz in the better-hearing ear compared to the ISO 7029:2017. The duration of deafness of the poorer-hearing ear showed no relationship with the hearing threshold of the better-hearing ear. The hearing threshold was significantly higher in typically bilaterally presenting etiologies (chronic otitis media, otosclerosis, and congenital hearing loss), except for Menière’s disease. Second, subjects that developed AHL did so in 5.19 ± 5.91 years and showed significant reduction in monosyllabic word and number recognition.

**Conclusions:**

Individuals with SSD show significantly poorer hearing in the better-hearing ear than individuals with NH from the ISO 7029:2017. In clinical practice, we should, therefore, inform our SSD patients that their disease is accompanied by a reduced hearing capacity on the contralateral side, especially in certain etiologies.

## Introduction

Single-sided deafness (SSD) is defined as severe to profound hearing loss in the poorer-hearing ear and (almost) normal-hearing (NH) in the better-hearing ear.

In a previous study we have shown, that the better-hearing ear of subjects with SSD has a significantly higher air-conducted (AC) pure-tone average in the frequencies 0.5, 1, 2, and 4 kHz (AC PTA4) compared with age- and gender-controlled hearing thresholds from ISO 7029:2017 [3].

One possible explanation for the higher AC PTA4 is an increased strain on the last-hearing ear, as persons with SSD tend to turn their better-hearing ear towards sound sources. This idea is supported by high-tone accentuated hearing impairment, such as the hearing impairment seen in persons with presbyacusis. To check this hypothesis, we included hearing thresholds from 0.125 kHz up to 8 kHz in the present study. In addition, we included monosyllabic word recognition and number recognition to evaluate the impact on speech recognition.

In our prior study, treatment with a cochlear implant (CI), duration of deafness, and etiology showed no significant relationship with the hearing ability of the better-hearing ear [3]. It was surprising that etiology showed no significant relationship, considering frequently bilateral occurring diseases, such as otosclerosis [4–10], Menière’s disease [11–13], and enlarged vestibular aqueduct syndrome [14]. We hypothesized that selection bias (only patients requested CI treatment were enrolled) might be a contributing factor. In the present study, we, therefore, acquired hearing results from individuals with SSD and asymmetric hearing loss (AHL) with and without CI from in- and out-patient care to incorporate earlier stages of the disease and to improve the charting of the disease over time.

In the present study, we also included measurements of tinnitus burden. We suspected a relationship between tinnitus burden and hearing capacity, because Mertens et al. [15] described a significant influence of tinnitus in the poorer-hearing ear on speech recognition in background noise in the better-hearing ear in subjects with SSD.

## Methods

The present study was performed with approval of the Ethics Commission Freiburg (No. 560-19) and in compliance with national law and the Declaration of Helsinki of 2013 (in the current revised edition) (DRKS00022115).

### Study participants

We recruited subjects (over 18 years) with SSD and AHL that presented at one of the participating hospitals: University Hospital Freiburg, Central Army Hospital Koblenz, Medical University Hanover, and University Hospital Marburg.

Subjects with SSD and AHL had severe to profound hearing loss in the poorer-hearing ear and differed in the hearing capacity of the better-hearing ear: SSD had an untreated AC PTA4 ≤ 30 dB HL and AHL an untreated AC PTA4 > 30 dB HL to ≤ 60 dB HL. In SSD the interaural asymmetry was required to be ≥ 30 dB HL [1].

### Data acquisition

We searched the databases of all participating hospitals for candidates. After receiving informed consent and releases from confidentiality, we contacted primary doctors of otorhinolaryngology to receive auditory measurements performed before and after consultation at the participating hospitals.

### Acquired data

Anamnestic data included age, gender, duration of deafness, treatment with CI, and etiology. We did not acquire data with regard to alternative treatment strategies, such as Contralateral Routing of Signals (CROS) or bone-anchored hearing system (BAHS). In non-CI users we calculated duration of deafness from the anamnestic onset of deafness to the date of measurement. For CI users the duration of deafness preoperatively was defined from the anamnestic onset of deafness to the date of measurement. After CI surgery deafness duration remained the duration between anamnestic onset of deafness and CI surgery. Etiology was arranged into eleven categories: (1) sudden hearing loss (SHL), (2) trauma (cranio-cerebral trauma with contusion labyrinthi, petrous bone fracture), (3) vestibular schwannoma, (4) Menière’s disease, (5) infectious disease (meningitis, mumps, labyrinthitis, influenza, acute otitis, neurolues, scarlet fever, measles) (6) chronic otitis media (OM), (7) congenital hearing loss, (8) otosclerosis, (9) post-ear-surgery (post-op) hearing loss, (10) other (large aquaeductus syndrome, Cogan-1-syndrom, Von Hippel–Lindau-Syndrome, status after radiotherapy, status after brain stem ischemia), and (11) unknown (no routine genetic screening).

If available, we retrospectively included bone-conducted (BC) and AC thresholds of both ears for the frequencies 125 Hz to 8 kHz and monosyllabic word recognition and number recognition using the Freiburg intelligibility test [17] from the participating hospitals and private practice otorhinolaryngologists.

To evaluated tinnitus burden we prospectively collected numerical rating scale (NRS) between 0 and 10 (10 representing the highest tinnitus burden [18]) and the tinnitus questionnaire by Goebel and Hiller [19].

We compared the hearing threshold with hearing thresholds from the ISO 7029:2017, which defines hearing thresholds for female and male “otologically normal persons” between 20 and 80 years. “Otologically normal person” is defined as an adult without symptoms of ear disease, complete obstruction of the auditory canal, excessive noise exposure, contact with ototoxic substances, and hereditary hearing loss. Hearing thresholds for the ISO 7029:2017 were retrieved by presenting a pure tone via a headphone to one ear. To compare subjects with SSD included in the present study we produced a “control group” consisting of hearing thresholds from subjects with same aged and gender derived from the ISO 7029:2017.

### Data analysis

Statistical data analysis was performed with Gnu R. The Shapiro–Wilk test showed non-normal distribution, so we applied Wilcoxon signed rank tests and Kruskal–Wallis rank sum tests (Table 1). For correlations, we used Pearson’s correlation analysis.

**Table 1:** Potential influencing factors, used statistical test and results

Audiological test	Potential influencing factor	Number of participants	Number of measurements	Statistical test	Results
AC pure-tone thresholds	Participants with SSD vs. NH cohort (Fig. 1)	SSD = 277	*n* = 1933	Wilcoxon signed rank test	0.125 kHz, 0.5 kHz, 0.75 kHz, 1 kHz, 1.5 kHz, 2 kHz, 3 kHz, 4 kHz, 6 kHz, 8 kHz: *p* < 0.001***
	CI user vs. non-CI user	CI = 178 (64%) Non-CI = 99 (36%)	–	–	–
	CI user (preop.) vs. non-CI user	CI = 163 Non-CI = 99	CI = 700 Non-CI = 433	Wilcoxon signed rank test	0.125 kHz: *p* < 0.05* 8 kHz: *p* < 0.01** 0.5 kHz, 0.75 kHz, 1 kHz, 1.5 kHz, 2 kHz, 3 kHz, 4 kHz, 6 kHz: n.s
	CI user (postop.) vs. non-CI user (Fig. 5)	CI = 101 Non-CI = 99	CI = 800 Non-CI = 433	Wilcoxon signed rank test	0.5 kHz, 0.75 kHz, 1 kHz, 1.5 kHz, 2 kHz, 3 kHz, 4 kHz: *p* < 0.001*** 6 kHz: n.s
AC PTA4	CI user (preop.) vs. non-CI user	CI = 155 Non-CI = 99	CI = 692 Non-CI = 435	Wilcoxon signed rank test	n.s
	CI user (postop.) vs. non-CI user	CI = 101 Non-CI = 99	CI = 798 Non-CI = 435	Wilcoxon signed rank test	*p* < 0.01**
	Duration after CI (Fig. 6)	Preop.. = 121 Postop.: 3 months = 86 6 months = 105 1 year = 99 2 years = 69 3 years = 28 4 years = 28 5 years = 22	Preop.. = 121 Postop.: 3 months = 86 6 months = 105 1 year = 99 2 years = 69 3 years = 28 4 years = 28 5 years = 22	Kruskal–Wallis rank sum test	n.s
AC PTA4 difference	Etiology (Fig. 3)	SSD = 277	*n* = 1933	Kruskal–Wallis rank sum test	*p* < 0.001***
	Duration of deafness (Fig. 2)	SSD = 264 (95%)	*n* = 1849	Pearson’s correlation analysis	*p* < 0.001***; cor = 0.08
	Tinnitus—NRS	*n* = 113 (41%)	*n* = 113	Pearson’s correlation analysis	n.s
	Tinnitus—tinnitus questionnaire	*n* = 171 (62%)	*n* = 171	Pearson’s correlation analysis	n.s
Number recognition	CI user vs. non-CI user	CI = 149 Non-CI = 91	CI = 595 Non-CI = 185	Wilcoxon signed rank test	n.s
Monosyllabic speech recognition	CI user vs. non-CI user	CI = 151 Non-CI = 90	CI = 609 Non-CI = 181	Wilcoxon signed rank test	n.s
	Etiology	n = 241 (87%)	n = 790	Kruskal–Wallis rank sum test	*p* < 0.001***
Tinnitus loudness	CI user vs. non-CI user	CI = 15 Non-CI = 5	CI = 15 Non-CI = 5	Wilcoxon signed rank test	n.s
Tinnitus frequency	CI user vs. non-CI user	CI = 15 Non-CI = 5	CI = 15 Non-CI = 5	Wilcoxon signed rank test	n.s
Tinnitus—NRS without CI	CI user vs. non-CI user	CI = 88 Non-CI = 25	CI = 88 Non-CI = 25	Wilcoxon signed rank test	*p* < 0.01**
Tinnitus—questionnaire	CI user vs. non-CI user	CI = 110 Non-CI = 61	CI = 110 Non-CI = 61	Wilcoxon signed rank test	n.s

Level of significance: *** - *p* < 0.001; ** - *p *< 0.01; * *p* < 0.05

To compare groups with significant differences in age and/or gender distribution, we employed the AC PTA4 difference as the dependent variable. The AC PTA4 difference is the age- and gender-corrected difference between the individual AC PTA4 of the better-hearing ear of subjects with SSD and hearing thresholds from ISO 7029:2017 (PTA4 better-hearing ear—PTA4 from ISO 7029:2017). AC PTA4 differences > 0 indicate that the AC PTA4 of the subjects with SSD is greater than the AC PTA4 from ISO 7029:2017. If the AC PTA4 difference is = 0 there is no difference between the subjects with SSD and the ISO 7029:2017 control group. We applied the AC PTA4 difference only to compare subjects with SSD, as in subjects with AHL a mild to moderate hearing loss on the better-hearing side is prevalent, and therefore, an age and/or gender correction with the ISO 7029:2017 is not applicable.

We categorized our participants into three groups: (1) AHL in all included measurements, (2) SSD in all included measurements, and (3) hearing threshold of the better-hearing ear deteriorated from SSD to AHL during the study period. To test this categorical variable, we used a Chi-squared test.

## Results

We enrolled 323 subjects from which 277 were subjects with SSD and 46 subjects with AHL (Table 2).

**Table 2:** Details of enrolled study participants

	Subjects with SSD (includes subjects that went from SSD to AHL)	Subjects that went from SSD to AHL	Subjects with AHL
*n*	277	104	46
Hospital	University Hospital Freiburg: 284 University Hospital Marburg: 19 Central Army Hospital Koblenz: 18 Medical University Hannover: 2		
Gender	♀ 153 ♂ 124	♀ 52 ♂52	♀ 22 ♂ 24
Poorer-hearing ear	Right: 129 Left: 148	Right: 57 Left: 47	Right: 24 Left: 22
CI	CI = 178 Non-CI = 99	CI = 74 Non-CI = 30	CI = 31 Non-CI = 15
Duration of deafness in the poorer-hearing ear (years)	6.88 ± 10.72	9.33 ± 12.55	13.04 ± 12.68
AC PTA4 of the better-hearing ear (dB HL)	14.72 ± 6.83	SSD: 21.12 ± 12.27 AHL: 35.54 ± 19.66	33.70 ± 16.72
Etiology			
Sudden hearing loss	97	34	17
Vestibular schwannoma	31	8	4
Trauma	18	8	3
Menière’s disease	18	8	0
Infectious disease	15	3	4
Chronic otitis media	13	6	1
Congenital	10	3	2
Otosclerosis	7	3	2
Post-ear-surgery	6	2	1
Other	6	4	3
Unknown	56	25	9

Etiology group “Infectious disease” (n): meningitis (6), mumps (6), labyrinthitis (3), influenza otitis (1), neurolues (1), scarlet fever (1), measles (1) Etiology group “Other” (n): progressive hearing loss (4), large aquaeductus syndrome (1), Cogan-1 syndrome (1), Von Hippel–Lindau syndrome (1), status after radiotherapy (1), status after brain stem ischemia (1)

### SSD

#### Pure tone-audiometry

**Fig. 1:**
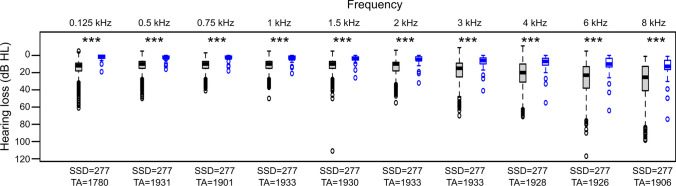
Comparison of air-conducted pure-tone thresholds from 0.125 kHz to 8 kHz between subjects with SSD (grey) and age- and gender-controlled hearing thresholds from ISO 7029:2017 (blue). *SSD* subjects with single-sided deafness; level of significance: *** - *p* < 0.001; ** - *p *< 0.01; * *p* < 0.05

From 277 subjects with SSD, we could include 1933 AC pure-tone audiograms and 1572 BC pure-tone audiograms (Table 1). Comparing the AC pure-tone thresholds from subjects with SSD to age- and gender-controlled hearing thresholds from ISO 7029:2017 we saw a significantly higher hearing thresholds in subjects with SSD at each measured frequency (Table 1, Fig. 1).

To check for systemic error, we compared the subjects with SSD from the hospitals Freiburg, Marburg, and Koblenz separately. Each analysis revealed significantly higher thresholds for subjects with SSD in each measured frequency (*p* < 0.05). A separate analysis of the Hannover group was not possible, because only two patients participated.

To check for the influence of an air–bone gap, we performed separate tests, including only thresholds with an air–bone gap smaller than 10 dB (excluding 0.125 and 8 kHz). We were able to retrieve air–bone gaps from 1572 pure-tone audiograms, because in 1572, a BC pure-tone audiogram was available. 249 subjects showed an air–bone gap smaller than 10 dB in 1332 audiograms. These analyses also revealed significantly higher hearing thresholds at each frequency (*p* < 0.05).

#### Duration of deafness

**Fig. 2:**
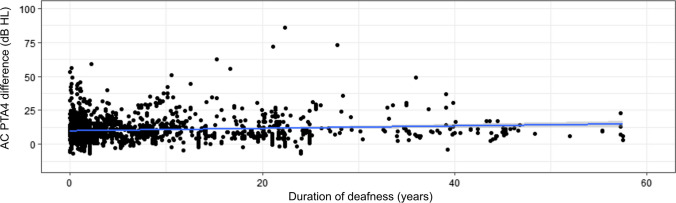
Correlation of air-conducted pure-tone thresholds average difference (AC PTA4 difference = PTA4 better-hearing ear—PTA4 from ISO 7029:2017) with duration of deafness in years in subjects with SSD. The AC PTA4 difference is the difference between the individual AC PTA4 of the better-hearing ear of subjects with SSD and age- and gender-controlled hearing thresholds from ISO 7029:2017. *SSD* subjects with single-sided deafness

In most subjects with NH (ISO 7029:2017) the hearing threshold increases over the life span. When investigating the effect of duration of deafness we have to correct for this natural increase in hearing threshold. We, therefore, used the age- and gender-corrected AC PTA4 difference (PTA4 better-hearing ear—PTA4 from ISO 7029:2017) to investigate the relationship between deafness duration and hearing capacity of the better hearing ear. We, therefore, derived an age- and gender-corrected AC PTA4 difference correlated with the duration of deafness with hearing performance in the better-hearing ear (Table 1). The AC PTA4 difference showed a significant positive correlation (*p* < 0.001; cor = 0.08) with the duration of deafness (Fig. 2).

#### Etiology

Age and gender were significantly different between various etiology groups. As we cannot control number and monosyllabic word recognition for the bias of age and gender, we did not compare number and monosyllabic word recognition between etiology groups. In case of hearing thresholds, we used the age- and gender-corrected AC PTA4 difference (PTA4 better-hearing ear—PTA4 from ISO 7029:2017) to investigate the relationship between etiology and hearing capacity of the better-hearing ear in subjects with SSD. We saw a significant relationship in the Kruskal–Wallis rank sum test between etiology and AC PTA4 difference (Table 1). In the post-hoc analysis we excluded the etiology groups “others” and “unknown”.

**Fig. 3:**
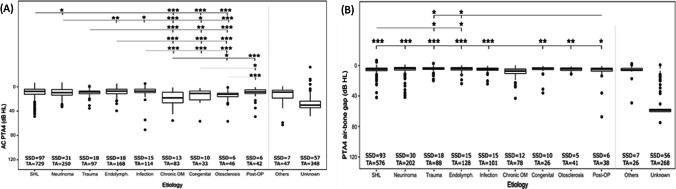
**A** Comparison of the average difference in air-conducted pure-tone thresholds (AC PTA4 difference = PTA4 better-hearing ear—PTA4 from ISO 7029:2017) in relation to etiology in subjects with SSD. The AC PTA4 difference is the difference between the individual AC PTA4 of the better-hearing ear of subjects with SSD and age- and gender-controlled hearing thresholds from ISO 7029:2017. **B** Comparison of the average difference in air–bone gap pure-tone thresholds (AC PTA4 air–bone gap = PTA4 better-hearing ear—PTA4 from ISO 7029:2017) in relation to etiology in subjects with SSD. *SSD* subjects with single-sided deafness, *TA* pure-tone measurements, *SHL* sudden hearing loss, *Schwannoma* vestibularis schwannoma, *chronic OM* chronic otitis media; level of significance: *** - *p* < 0.001; - ** *p* < 0.01; * - *p* < 0.05

Subjects with chronic otitis media (OM) showed the highest AC PTA4 difference of all etiology groups (Fig. 3A). To investigate the impact of conductive hearing impairment, we compared air–bone gaps between etiologies in cases in which BC PTA4 was available. Subjects with chronic OM had a significantly higher PTA4 air–bone gap compared with all other etiologies. (Fig. 3B).

#### CI user vs. non-CI user

We found no significant difference in age and gender between CI users and non-CI users. We could, therefore, compare number recognition, monosyllabic word recognition, AC PTA4, preoperative and postoperative thresholds without bias of age or gender.

**Fig. 4:**
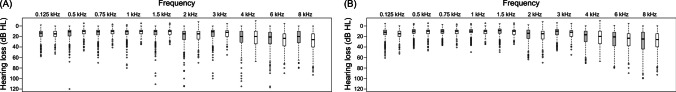
Comparison of air-conducted pure-tone thresholds from 0.125 kHz to 8 kHz between non-CI users with SSD (white) and **A** preoperative pure-tone audiograms of CI users with SSD (grey) and **B** postoperative pure-tone audiograms of CI users with SSD (grey). *SSD* subjects with single-sided deafness; level of significance: *** - *p* < 0.001; ** - *p* < 0.01; * - *p* < 0.05

In CI users, preoperative AC pure-tone thresholds of all frequencies from 0.125 to 8 kHz in the better-hearing ear were significantly lower than in non-CI users (Table 1, Fig. 4A). The postoperative AC pure-tone thresholds were significantly lower in CI-users than in no-CI users for the frequencies from 0.125 to 4 kHz and not for 6 kHz and 8 kHz (Table 1, Fig. 4B).

Preoperative AC PTA4 were available in 692 of 700 AC pure-tone audiograms, as the value for 0.5, 1, 2, or 4 kHz was missing in 8 AC pure-tone audiograms. Preoperative AC PTA4 did not differ significantly between CI users and non-CI users (Table 1). Postoperative AC PTA4 was significantly lower in CI users (Table 1).

With regard to the CI users, we included measurements of number recognition and monosyllabic word recognition of the better-hearing ear performed before and after CI, in the statistical analysis. Wilcoxon signed rank tests revealed no significant difference between CI users and non-CI users (Table 1).

To investigate changes in the better-hearing ear after the CI operation, we compared AC PTA4 results preoperative up to 5 year postoperative. No significant differences between the included appointments were revealed (Table 1).

#### Tinnitus NRS and tinnitus questionnaire (German adaptation by Goebel and Hiller)

We correlated the (1) NRS and (2) questionnaire results with the AC PTA4 difference. Neither correlation was significant (Table 1).

CI users had a significantly higher NRS than non-CI users (*p* < 0.05). A significantly lower NRS was found in CI users while using the CI than without using the CI (*p* < 0.001).

### SSD to AHL

The categories (1) AHL in all measurements (*n* = 46), (2) SSD in all measurements (*n* = 173), and (3) SSD to AHL (*n* = 104) did not differ significantly in gender, treatment with CI, or etiology.

Subjects that went from SSD to AHL during the included measurements (Table 2) went from AC PTA4 of 21.12 ± 12.27 dB HL to 35.54 ± 19.66 dB HL. Monosyllabic word recognition went from 88.10% ± 20.40% at 65 dB to 73.79% ± 27.80% at 65 dB (*p* < 0.001). The 50% correct numeric speech recognition went from 14.52 ± 10.02 dB SPL to 22.80 ± 27.80 dB SPL (*p* < 0.001).

**Fig. 5:**
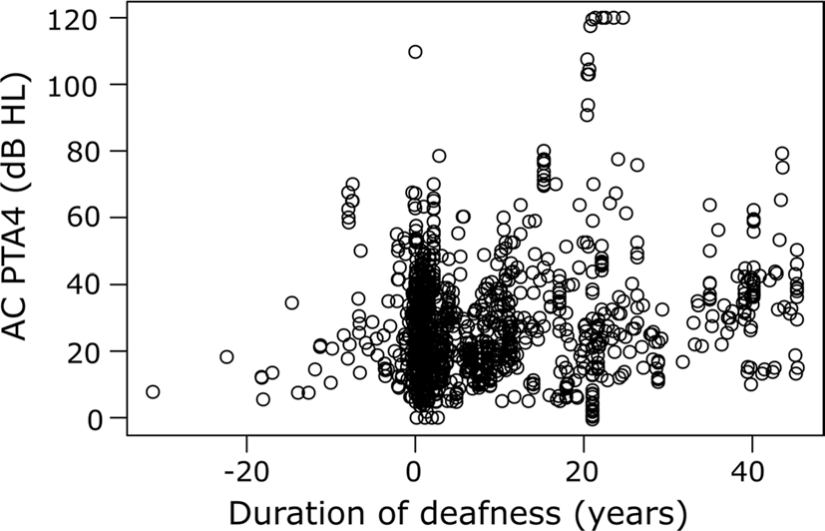
Correlation of air-conducted pure-tone thresholds average (AC PTA4) with duration of deafness in years in subjects that went from SSD to AHL. *AHL* asymmetric hearing loss, *SSD* subjects with single-sided deafness

On average the subjects went from SSD to AHL at 5.19 ± 5.91 years (median: 2.78 years) after the first included measurement (Fig. 5). In cases of fluctuating hearing thresholds in the better-hearing ear we measured the time from SSD to AHL from the first included measurement diagnosed with SSD to the measurement after which all hearing threshold were diagnosed with AHL. In a Kruskal–Wallis rank sum tests the etiology had no relationship with the duration between the diagnosis of SSD and AHL.

## Discussion

We found a significant higher hearing threshold in the better hearing-ear of subjects with SSD compared with age- and gender-controlled hearing thresholds from ISO 7029:2017. Subjects that developed AHL did so in 5.19 ± 5.91 years and showed significant reduction in monosyllabic word recognition and numeric speech recognition. Age, gender and treatment with CI were not correlated with developing AHL.

The difference between ISO 7029:2017 and subjects with SSD through all frequencies might be a systematic error. However, this is unlikely, because we included audiograms from 192 subjects performed by 156 private practice otorhinolaryngologists who performed their hearing measurements independently of each other. To confront this possible bias, we analyzed subjects from each participating hospital. We saw a significantly higher hearing threshold in all frequencies for the better-hearing ear of subjects with SSD in each separate analysis. The large number of independent investigators and the separate analyses performed minimize the possibility of a systematic bias substantially, enabling us to be confident in the present results.

The higher hearing threshold in the better-hearing ear of participants with SSD is more pronounced at higher frequencies, but the hearing loss is evident over all frequencies. This result opposes our hypothesis that individuals with SSD strain their last-hearing ear by turning it towards sound sources. This strain could result from additional noise and/or stretching of the cervical vessels resulting in reduced blood flow. This idea is also weakened by the low correlation between AC PTA4 difference (controlled for age) and duration of hearing impairment. If early alteration because of hearing stress on the cochlea caused higher hearing threshold, we would expect a strong correlation with the duration of hearing impairment and/or a correlation to inner ear trauma because of noise exposure. Similarly, AC PTA4 difference of the better-hearing ear did not change significantly in SSD CI users preoperatively to 5 year post-CI. The duration of deafness was not significantly different between subjects with SSD, subjects with AHL and subjects that went from SSD to AHL. In subjects that went from SSD to AHL hearing loss in the better-hearing ear progressed in mean within the first 5 years. Interestingly, the etiology showed no relation with the duration of progression from SSD to AHL.

We choose to investigate CI as a potential factor, because a CI would reduce the turning of the last-hearing ear towards sound sources. Number and monosyllabic word recognition did not differ significantly between CI users and non-CI users, because number and monosyllabic word recognition of the better-hearing ear showed a ceiling effect at 100% correct. To investigate, if hearing thresholds already differed before implantation, we compared preoperative thresholds with thresholds from non-CI users; we only found a significant higher hearing threshold in non-CI users at the frequencies 125 Hz and 8 kHz. Therefore, we have no selection bias in the frequencies from 0.5 to 6 kHz when comparing postoperative hearing thresholds with hearing thresholds from non-CI users. After treatment with CI, we saw significantly lower hearing thresholds in CI users with SSD from 0.5 to 4 kHz. Differences in socio-economic status, personal support system, educational background, noise exposure in the work place and intelligence could contribute to these differences. Due to the retrospective nature of the study, we did not investigate these factors. Interestingly, the lower hearing threshold in CI users is evident over all frequencies with the exception of 6 kHz. This also contradicts our hypothesis.

To investigate this hypothesis further, head movements towards sound sources by individuals with SSD can be monitored, and persons with higher and lower noise exposure can be compared. In addition, hearing loss at frequencies higher than 8 kHz can be investigated in the better-hearing ear of subjects with SSD. The hearing impairment over all frequencies of the better-hearing ear suggests that it might be caused by sympathetic hearing loss. Similar to sympathetic ophthalmia, the hearing loss is caused by damage to the contralateral cochlear resulting in an autoimmune response to the contralateral side [20–22].

BC thresholds reflect the hearing capacity of the cochlear and auditory nerve. However, BC thresholds do not reflect the actual hearing capacity in everyday life. In the present study, unlike in our previous study on this subject [3], we compared the AC threshold. We choose to do so for two reasons. (1) We compared our participants with ISO 7029:2017, which only contains AC thresholds. (2) As we included measurements from private practice otorhinolaryngologists, BC thresholds were not regularly available. This choice led to a challenge: conductive hearing impairment causing air–bone gaps higher than 10 dB might influence our results (the reason that we choose BC thresholds in our previous study). We, therefore, excluded all measurements with an air–bone gap ≥ 10 dB and reran the comparison between subjects with SSD and hearing thresholds from ISO 7029:2017 for each frequency. These analyses also revealed a significantly higher hearing thresholds in subjects with SSD in each frequency. Therefore, conductive hearing loss did not contribute to the higher hearing threshold in the better-hearing ear of subjects with SSD. A limitation is, as described above, that air–bone gaps were only available in audiograms with additionally measured BC thresholds.

However, we found that subjects with chronic OM showed the highest AC PTA4 difference, because subjects with chronic OM had the largest air–bone gap in the better-hearing ear compared to other etiologies. The elevated air–bone gap is most likely caused by reduced middle ear ventilation on both sides. The contralateral ear in patients with chronic OM show abnormalities in the otoscopic examination from 50% up to 83.3% [23, 24]. Bilateral chronic OM is seen in 12% of cases [25].

Individuals with congenital SSD had an elevated hearing threshold compared with other etiologies in the present study. Two explanations can be proposed: (1) the neurological influence of congenital SSD and (2) the etiology of congenital SSD. Unilateral hearing in the vulnerable phase before the age of 4 years can lead to central reorganization with long-lasting effects [26–28]. In the literature, congenital SSD is reported to be caused by congenital CMV infection in over 20% of children with SSD [28–30]. This is relevant, as up to 75% of children with SSD attributable to congenital CMV develop delayed-onset contralateral hearing loss [31]. In addition, alterations in MRI and CT might be more frequent on the contralateral side in individuals with congenital SSD than in NH individuals [32]. Future studies including the systematic evaluation of MRI and CT data would thus be of interest.

Subjects with otosclerosis also revealed higher AC PTA4 differences than other etiologies. A bilateral otosclerosis is seen in 62–80% individuals and usually develops in one ear first [4–10]. Therefore, our findings are in agreement with the literature.

Surprisingly, the included subjects with Menière’s disease showed no significantly higher hearing thresholds than other etiologies. From the literature we know that Menière’s disease occurs bilaterally in approximately one-third of cases [11–13], and that, after 20 years of disease duration, over 40% of subjects develop bilateral Menière’s disease [33, 34]. In our study, only two subjects with Menière’s disease had had a duration of hearing loss longer than 20 years. Eight out of 18 subjects progressed from SSD to AHL during the time span of the included hearing measurements. The reason that we have no significantly increased hearing threshold in subjects with Menière’s disease might be, because we were only able to include two subjects with a longer duration of disease. Perhaps some of the included participants will develop bilateral disease during their lifetime.

We did not compare the recognition of numbers and monosyllabic words between the etiologies, because the various etiological groups differed significantly in age and gender. Since we could not control for age and gender, in contrast to the AC PTA4 difference, the results of the speech recognition tests might possibly be overshadowed by the effect of age or gender.

Similarly, neither NRS of tinnitus burden nor results from the tinnitus questionnaire after Goebel and Hiller [19] showed a significant relationship with the hearing ability of the better-hearing ear in subjects with SSD. This might be, because in contrast to Mertens et al. [15], we were not able to included speech recognition in background noise in our retrospective study design. In future prospective studies, an investigation of the relationship between tinnitus and more challenging hearing measurements, such as speech recognition in background noise or localization of sound sources, would be of interest.

In clinical practice, we should inform our SSD patients that their disease is accompanied by a reduced hearing capacity on the contralateral side, especially in certain etiologies (congenital SSD, otosclerosis and chronic otitis media), and that a longer SSD duration and tinnitus will not worsen their contralateral ear. CI treatment showed no negative relationship with the hearing threshold of the contralateral better-hearing ear.

## Data Availability

The datasets analyzed during the current study are available from the corresponding author on reasonable request.
